# Anti-Angiogenic Effects of Phytochemicals on miRNA Regulating Breast Cancer Progression

**DOI:** 10.3390/biom10020191

**Published:** 2020-01-27

**Authors:** Elizabeth Varghese, Alena Liskova, Peter Kubatka, Samson Mathews Samuel, Dietrich Büsselberg

**Affiliations:** 1 Department of Physiology and Biophysics, Weill Cornell Medicine-Qatar, Education City, Qatar Foundation, Doha P.O. Box 24144, Qatar; elv2007@qatar-med.cornell.edu (E.V.); sms2016@qatar-med.cornell.edu (S.M.S.); 2 Clinic of Obstetrics and Gynecology, Jessenius Faculty of Medicine, Comenius University in Bratislava, 03601 Martin, Slovakia; alenka.liskova@gmail.com; 3 Department of Medical Biology, Jessenius Faculty of Medicine, Comenius University in Bratislava, 03601 Martin, Slovakia; kubatka@jfmed.uniba.sk

**Keywords:** tumor angiogenesis, angiomiRs, phytochemicals, endothelial cell metabolism

## Abstract

Several phytochemicals have been identified for their role in modifying miRNA regulating tumor progression. miRNAs modulate the expression of several oncogenes and tumor suppressor genes including the genes that regulate tumor angiogenesis. Hypoxia inducible factor-1 alpha (HIF-1α) signaling is a central axis that activates oncogenic signaling and acts as a metabolic switch in endothelial cell (EC) driven tumor angiogenesis. Tumor angiogenesis driven by metabolic reprogramming of EC is crucial for tumor progression and metastasis in many different cancers, including breast cancers, and has been linked to aberrant miRNA expression profiles. In the current article, we identify different miRNAs that regulate tumor angiogenesis in the context of oncogenic signaling and metabolic reprogramming in ECs and review how selected phytochemicals could modulate miRNA levels to induce an anti-angiogenic action in breast cancer. Studies involving genistein, epigallocatechin gallate (EGCG) and resveratrol demonstrate the regulation of miRNA-21, miRNA-221/222 and miRNA-27, which are prognostic markers in triple negative breast cancers (TNBCs). Modulating the metabolic pathway is a novel strategy for controlling tumor angiogenesis and tumor growth. Cardamonin, curcumin and resveratrol exhibit their anti-angiogenic property by targeting the miRNAs that regulate EC metabolism. Here we suggest that using phytochemicals to target miRNAs, which in turn suppresses tumor angiogenesis, should have the potential to inhibit tumor growth, progression, invasion and metastasis and may be developed into an effective therapeutic strategy for the treatment of many different cancers where tumor angiogenesis plays a significant role in tumor growth and progression.

## 1. Introduction

Plants have been an integral part of traditional medicine. Natural compounds are gaining attention because of their potential to cure a variety of ailments, including cancer. Some secondary metabolites from plants inhibit tumor growth by interfering with tumorigenic signaling pathways. Cancer is defined by underlying principles called “hallmarks”, which are: a) sustained proliferation, b) inhibition of apoptosis, c) immune evasion, d) genomic instability, e) modified cellular energetics, f) sustained angiogenesis, g) invasion and metastasis, and h) evade growth suppression. The strategy for any anti-cancer therapy is to target any of the above principles. In this review we focus on miRNAs regulating tumor endothelial cell (EC) metabolism, EC angiogenic signaling and the natural compounds modulating angiogenic miRNA. We briefly describe the angiogenic signal transduction pathways involved in tumor endothelial cell (TEC) and metabolic pathways that drive angiogenic signaling in TEC. We also discuss the gaps in this research area, strategies and scope of targeting the energy metabolism in order to stop tumor angiogenesis.

## 2. Tumor Angiogenesis

Angiogenesis involves a myriad of events including extra cellular matrix remodeling, proliferation and migration of EC cells leading to formation of new blood vessels. Angiogenesis is an essential step to breast cancer progression and metastasis [1]. Earlier studies reported that tumor angiogenesis significantly correlated with the degree of micro vessel formation and aggressiveness of invasive breast carcinoma [2]. Increased angiogenic activity in breast pre-neoplastic lesions is related to poor prognosis [3,4]. Breast cancer cells direct the tumor angiogenesis via pro-angiogenic factors such as interleukin-1 (IL-1), interleukin-8 (IL-8), vascular endothelial growth factor (VEGF), basic fibroblast growth factor (bFGF), tumor necrosis factor α (TNFα) and matrix metalloproteinases 9 (MMP9) [5].

Angiogenesis is a tightly controlled process which is under the regulation of both activators and inhibitors. In normal tissue the angiogenic switch is turned off once the blood vessel formation is complete while in tumors this switch is continuously turned on. Tumor growth is angiogenesis dependent and tumors cannot grow beyond 1-2mm size without neovascularization [6,7]. Tumors progress from an avascular phase to vascular phase in order to invade and migrate. The phenotypic switch to vascular type is regulated by these chemicals called angiogenic factors which are secreted by the tumor cells, tumor associated macrophages and the stromal cells collectively called as the tumor micro environment (TME). They secrete tumor angiogenic factors (TAF) which recruit EC to form new blood vessels. The EC cells which are in a resting state switch to actively proliferating state under the influence of tumor angiogenic factors.

### 2.1. Angiogenic Signaling in EC

**Figure 1:**
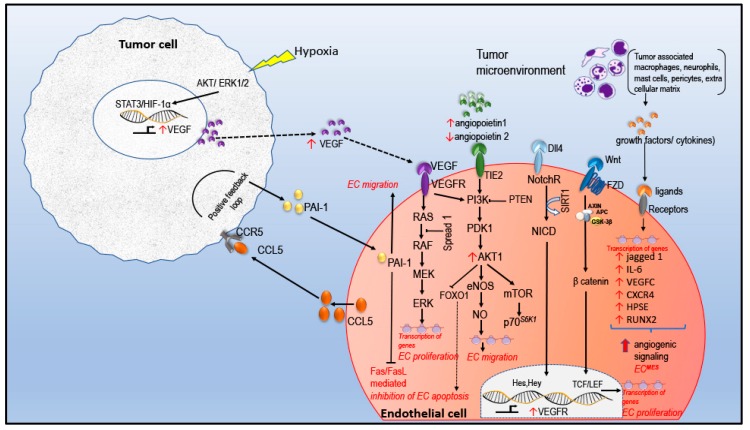
Illustration of tumor endothelial cell signaling. In tumor micro environment (TME), angiogenesis is mainly triggered by hypoxia which promotes generation of pro-angiogenic factors such as growth factors and cytokines by tumor cells and tumor associated stromal cells. Vascular endothelial growth factor/vascular endothelial growth factor receptor (VEGF/VEGFR) is the main axis of angiogenesis and hence is the most attractive target for anti-angiogenic treatment in cancer therapy. In invasive breast cancer (BC), VEGFR3 is up-regulated in tumor endothelial cell. Under low oxygen tension, transcription of HIF-1 α is increased which increases the synthesis of stress related proteins such as VEGF by tumor cells. Binding of factors to the endothelial cell (EC) receptors activates angiogenic signaling pathways mainly PI3K/AKT/mTOR/eNOS signaling. C-C motif chemokine ligand 5 (CCL5) a member of the cytokine family is detected in tumor samples. Increased plasminogen activator inhibitor-1 (PAI-1) secretion by the tumor cell up-regulates CCL5/CCR5 axis forming a +ve feedback loop leading to increased expression of transcription factors related to epithelial–mesenchymal transition (EMT). In addition, PAI-1 protects EC cells from Fas/Fas ligand (FasL) mediated apoptosis. Wnt signaling regulate angiogenesis via β catenin, as a result transcription factors TCF/LEF bind to promotor region of Wnt transcribed genes leading to EC cell proliferation and morphogenesis. The EC cells acquire mesenchymal phenotype in a TME, showing increased migratory, invasive and angiogenic property. Sustained EC cell signaling activates angiogenic process including proliferation, inhibition of apoptosis, migration, ultimately building the tumor vasculature [10,11,12].

EC are cells forming the endothelium, which lines the lumen of a blood vessel and is a metabolically active cell essential for the maintenance of vascular hemostasis which involves coagulation, fibrinolysis, platelet aggregation, vessel growth, vessel tonicity and vascular permeability [8]. EC of the normal tissue is continuous while tumor EC have an irregular shape and size with cytoplasmic extensions, projecting into the lumen creating gaps causing extravasation of fluid and cells into the surrounding space forming blood lakes [9]. Moreover, tumor EC exhibit altered metabolic and signaling pathways. Figure 1 depicts an overview of tumor EC signal transduction in angiogenesis.

When the tumor is deprived of nutrients and oxygen, it initiates a hypoxic stress response which mediates the expression of transcription factor hypoxia inducible factor (HIF). HIF-1α is up-regulated in many cancers. HIF-1α triggers the expression of hypoxia driven genes such as vascular endothelial growth factor (VEGF). Binding of HIF-1α and signal transducer and activator of transcription 3 (STAT3) to the promotor region of VEGF causes the maximum expression. VEGF is pro-angiogenic and VEGF/VEGFR axis form the primary axis of angiogenesis. VEGF drives recruitment of cells for angiogenesis and proliferation of endothelial cells. In addition, VEGF activates cytoskeletal re-arrangement and drives EC migration via an activated PI3K/AKT pathway [13]. Additional VEGF is secreted by pericytes under the positive regulation of platelet-derived growth factor (PDGF) secreted by the activated TEC. Following the binding of VEGF to VEGFR initiates different signaling cascade. VEGF induced activation of PI3K/AKT pathway bring about EC proliferation and EC survival via regulation of down-stream effectors, B-cell lymphoma 2 (Bcl-2), BCL2 associated agonist of cell death (BAD), p53. In other ways, AKT activation attenuates the intrinsic apoptotic pathways [14]. Apart from regulating apoptosis, AKT activation up-regulates eNOS and associated nitric oxide (NO) production, which also induces angiogenesis. NO also inhibits apoptosis, stimulates proliferation, invasion and metastasis [15]. Inhibition of NO blocks VEGF induced cell migration. Another aspect of hyper-activation of the AKT pathway is the inactivation of transcription factor Fork-head box class O (FOXO) whose target genes involves Bcl-2 interacting mediator of cell death (BIM), Fas ligand (FasL), p27, growth arrest and DNA damage (GADD45) that regulates apoptosis. Sustained activation of PI3K/AKT/mTOR pathway leads to formation of abnormal tumor vessels. Consequently, decreasing VEGF expression normalizes tumor vasculature [10]. EC in tumor microenvironment (TME) plays an active role in tumor growth and moreover a critical role in metastasis. Recently, Zhang et al. reported a novel plasminogen activator inhibitor-1 (PAI-1) and C-C motif chemokine ligand 5 (CCL5) signaling pathway that is a potential therapeutic target for TNBC patients [11]. They reported that EC cells are crucial in TNBC metastasis via PAI-1/CCL5 signaling pathway. In vitro studies revealed a positive feedback on PAI-1 and CCL5 axis in EC cells suggesting that PAI-1 could increase EC migration and angiogenesis (Figure 1). Analysis of tissue samples of TNBC patients revealed that PAI-1 correlates with angiogenesis, relapse and metastasis [11]. Wnt/beta-catenin signaling is important in breast cancer development as evident from immunohistochemical studies. Results show elevated levels of β-catenin and over-expression or down-regulation of specific Wnt proteins in 50% of breast carcinoma [16]. An activated Wnt signaling regulate cell fate by controlling cell proliferation, migration, apoptosis, angiogenesis and vessel re-modelling etc. [17]. Similarly, Notch signaling is another major signaling pathway in tumor angiogenesis. EC express both Notch receptors (Notch 1 and 4) and ligands (delta-like ligand 4 (DII4), Jagged 1) [18]. Evidence shows that DII4 is strongly expressed in tumor EC [18]. Jagged 1 expressed on the tumor cell has a positive effect on the Notch receptor on the EC, where the down stream signaling drives the transcription of target genes Hes and Hey [19]. In TME, EC cells are exposed to tumor derived activators which trigger EC to switch to a more active mesenchymal phenotype supporting invasion and metastasis [20]. This finding was supported by the work published by Ghiabi and co-workers, where they showed tumor induced EC^Mes^ phenotype contributes to tumor growth, survival, enhanced angiogenesis, stemness and invasiveness. In this study, they demonstrated the involvement of synergistic action of both Notch and TGF β pathway in the upregulation of several genes including Jagged1, Notch2, TGF β, epidermal growth factor receptor (EGFR), WNT5B, STAT2 in the EC^Mes^ phenotype [20]. Collectively, the tumor cell, TME and EC mutually support each other promoting tumor growth.

### 2.2. Tumor EC Metabolism in Regulating Tumor Angiogenesis

**Figure 2:**
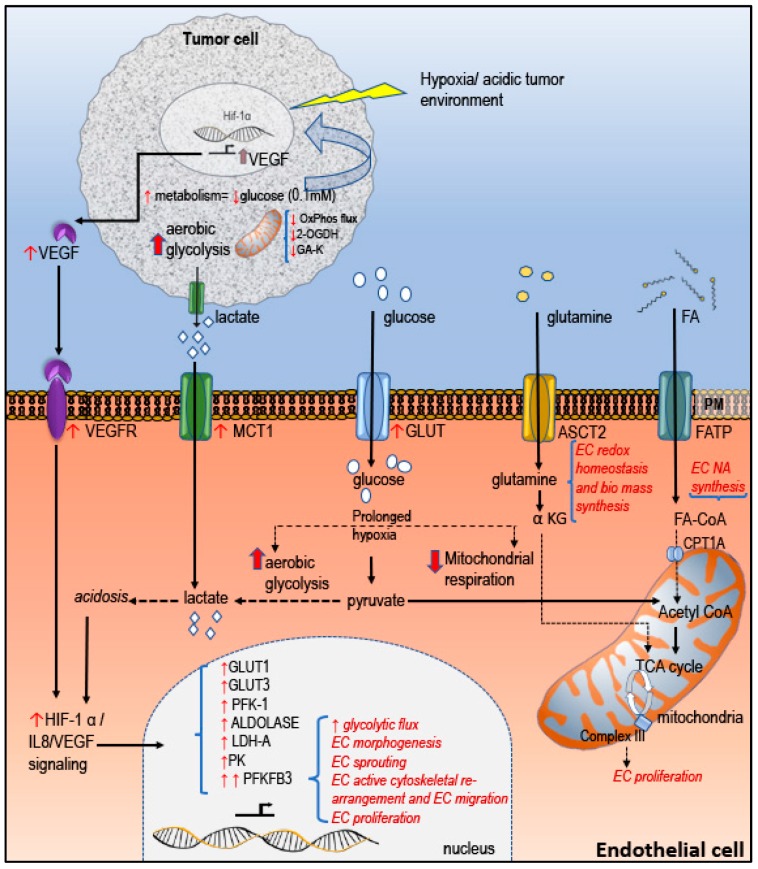
Angiogenesis in tumors is closely associated with EC metabolism. Glucose is the main energy resource of any cell. Glucose enters the EC cell through GLUT transporters (glucose transporters). Expression of GLUT transporters are up-regulated under hypoxic condition. Monocarboxylate transporter 1(MCT1), a transporter for lactate is highly expressed in cancers including breast. Hypoxia and acidic tumor environment turn “ON” the angiogenic switch. Under hypoxic condition OxPhos flux is reduced and the cells shift to a more aerobic glycolysis; as a result the pH of the cytoplasm becomes more acidic. Acidosis contributes to more robust expression of HIF-1α expression and increased IL8/VEGF signaling contributes to upregulation of glycolytic enzymes especially 6-phosphofructo-2-kinase/fructose-2,6-biphosphatase 3 (PFKFB3). PFKFB3 is essential for EC motility and sprouting. Two important biomolecules required for EC proliferation are glutamine and fatty acid (FA). FA entry into the mitochondria is facilitated via carnitine palmitoyl transferase 1A (CPT1A). FA oxidation is essential for nucleic acid synthesis and hence essential for EC proliferation. Glutamine is essential for redox homeostasis and biomass synthesis. In tumorigenesis, glutamine metabolism is up-regulated to compensate the energy requirement which was compromised due to reduced oxidative phosphor relation (OxPhos) flux [26,29,31,35,36,37].

An altered metabolism in tumor cells was first reported by Warburg [21,22]. In this seminal paper on altered tumor metabolism, Warburg observed a high rate of glucose utilization in tumors compared to its normal counterpart where glucose was converted to lactate rather than entering the mitochondria for oxidative phosphorylation; this phenomenon is termed the Warburg effect [23]. A recent review by Anne Teuwena et al. emphasizes and elaborates on the emerging role of EC metabolism in angiogenesis and lymphangiogenesis [24]. Normally, EC adapt to aerobic glycolysis irrespective of the oxygen available and generate up to 85% of their ATP through glycolysis [25]. Under the influence of pro-angiogenic factors EC phenotypically differentiate from a quiescent phalanx cell to migratory “Tip” cells and proliferative “Stalk cells” [26]. All the three phenotypically different EC diverge in cell signaling and metabolic activity [27]. Up-regulated glycolytic activity is attributed to high activity of 6-phosphofructo-2-kinase/fructose-2,6-biphosphatase 3 (PFKFB3) in EC (Figure 2). Findings regarding the differences in the normal EC and tumor EC indicate an up-regulated expression of glycolytic gene PFKFB3 and hence prove that tumor EC are highly glycolytic [28,29]. In experiments performed in mice with tumor, inhibition of PFKFB3 in EC reduced glycolysis, and subsequently reduced lactate level. Additionally, blocking PFKFB3 further reduced NF-κB signaling, invasion, metastasis, improved tumor perfusion and oxygenation by improving the integrity and stability of the vessels [28,30]. Moreover, blocking PFKFB3 improved the delivery and efficiency of the chemotherapeutic drug. Evidences support the assumption that this protein is a potential target for anticancer therapy. In addition to glycolysis, EC metabolism controls angiogenesis via an non-oxidative pentose-phosphate pathway (PPP), glutamine metabolism [31], FA metabolism [24,32]. PPP or hexose monophosphate shunt runs parallel to glycolytic pathway generates precursors for nucleic acid synthesis. PPP is found up-regulated in several cancers including breast, colon and prostate cancer [33,34].

## 3. Central Role of miRNA in the Regulation of Tumor Angiogenesis: The Role of Phytochemicals

Having discussed about angiogenesis in BC, especially in the context of invasion and metastasis, targeting angiogenic pathways is promising for the treatment of breast cancers. Though there are many pathways regulating angiogenesis, the key trigger is hypoxia induced activation of HIF-1α and up-regulated VEGF expression. In addition, ROS has complex role in angiogenesis. In both cases miRNAs play a regulatory role. miRNAs are important regulators of gene expression and dysregulation of miRNAs have been implicated in many disease such as cancers, cardiovascular diseases, and neurodisorders such as Alzheimer’s disease and Parkinson’s disease. miRNAs are 21–23 nucleotide long single stranded non-coding RNA that regulate gene expression post transcriptionally by either degrading or silencing the target mRNA, and thus cordinate cell physiology such as proliferation and apoptosis. Advances in miRNA studies identified their significant role in oncogenesis. miRNA profiling have shown that it varies from tumor to tumor. In the context of angiogenesis, miRNA fine tunes the angiogenic signaling in endothelial cells at various stages of angiogenesis. Some miRNAs are highly expressed and exclusive to endothelial cells such as miRNA 126. miRNAs can be pro- or anti-angiogenic.

Epidemiological studies reveal plant based dietary interventions markedly reduced BC risk and progression [38]. A growing body of literature shows that phytochemicals can regulate miRNA expression. Multiple studies highlight the anti-metastatic and anti-angiogenic properties of plant derived compounds where they target proliferation, inhibit secretion of MMP enzymes, growth factors such as VEGF and chemokines inducing metastasis [39].They can directly target the (BC relevant) miRNAs by transcriptional modification or epigenetic modification or by controlling miRNA processing [39]. However, up to date, most of the studies are limited to in vitro studies.

### 3.1. miRNA in EC VEGF Signaling

miRNAs that control every stages of oncogenesis and influence all the hallmarks of cancer are collectively grouped as oncomiRs. OncomiRs can target tumor suppressor proteins and they are generally found overexpressed, whilst tumor suppressor miRNAs are down-regulated in cancers. miRNA 140-5p, miRNA-34a, miRNA-145, miRNA-126 are some of the tumor suppressor miRNAs and miRNA-155, miRNA-21, miRNA-105, miRNA-9, miRNA-632 are found altered in BC [40,41]. Studies emphazise the regulatory role of miRNAs in angiogenesis which is an essential process for tumor growth and metastasis. The regulatory role of miRNA in angiogenesis was first reported in 2006 by Poliseno et al. [42,43]. This study investigated the role of miRNA in regulating angiogenesis related genes. Studies prove that miRNA can have dual effect on angiogenesis; miRNA that are pro-angiogenic and that are anti-angiogenic collectively termed as AngiomiRs [44]. The role of miRNA in angiogenesis was identified by knock down of two important enzymes in miRNA biogenesis; Dicer and Dorsha. Both in vivo and in vitro knock down experiments either decreased or induced a defective angiogenesis [45]. Emerging studies have shown that dysregulated miRNA is associated with tumor progression and tumor angiogenesis [45,46,47]. miRNA controls different aspects of BC angiogenesis and tumor progression by regulating apoptosis, proliferation, motility, energy metabolism, etc. More than forty miRNAs [48] were identified to be associated with tumor angiogenesis. Among the angiomiRs, miRNA-155, miRNA-153, miRNA-206, miRNA-467, miRNA-21, miRNA-34a and miRNA-126 respond to glucose level, miRNA-105, miRNA-206, miRNA-236 and miRNA-190 regulate metastasis, and miRNA-205, miRNA-206, miRNA-296, miRNA- 34a and miRNA-98 control proliferation and thus control endothelial physiology and tumor angiogenesis (Table 1).

AngiomiRs have multiple regulatory role in maintaining EC function. Altered miRNA expression is triggered following hypoxia, acidosis, VEGF stimulation and other tumor generated growth factors. In the angiogenic pathway VEGF signaling is a major contributor in angiogenesis. Apart from transcription factors, expression of VEGF and its receptors are also regulated at the miRNA level (i.e., at the post transcriptional level). Hunter et al. examined the miRNA expression in BC tissue samples and found positive correlation of angiogenesis/lymphangiogenesis marker with the altered miRNA expression [46]. Further invitro studies showed two miRNAs, miRNA-526 and miRNA-655 act via PI3K/AKT by targeting PTEN and EP4. Apart from angiogenesis, miRNA-526 and miRNA-655 are implicated in stemness, EMT, invasion and migration [46]. PTEN, an inhibitor of PI3K, is often associated with angiogenesis in different tumors [49]. Knock down experiments of PTEN increase the VEGF expression and increase the proliferation and migration of vascular endothelial cells [50]. In the VEGF signaling pathway, PTEN is another target of angiomiRs such as miRNA-21, miRNA-526b, miRNA-655 regulating angiogenesis via PI3K/AKT/VEGF/eNOS pathway.

As VEGF signaling is the primary signaling pathway promoting angiogenesis, the factors inducing VEGF expression and the downstream signaling following VEGF activation have potential targets for anti-angiogenic therapy. Activation of HIF-1, STAT3 [51] and production of NO [52], increased glycolytic flux [53] up-regulates VEGF expression [54,55]. Therefore, the miRNA regulating the expression of these three factors are attractive targets for anticancer therapy [56]. Angiogenic miRNA regulating VEGF expression are found dis-regulated in BC (Table 1). miRNA-206, miRNA-100, miRNA-20a, miRNA-140-5p, miRNA-126, miRNA-20a, miRNA-153, miRNA-205, miRNA-497, miRNA-145, miRNA-29 and miRNA-23a are AngiomiRs directly or indirectly targeting VEGF signaling. Previous studies reported that miRNA-205 has a tumor suppressor role. Hu et al. reported that miRNA-205 directly binds to 3′-UTR of VEGFA and FGF2 transcripts and down-regulated mRNA expression in BC patients [57]. miRNA-126 which is exclusive to EC mediates vessel integrity in vivo and promote the pro-angiogenic activity of VEGF and FGF by repressing Spread 1 and PIK3R2 [58]. It is interesting to note that VEGFA and miRNA-126 have an inverse relation, and thereby act as tumor suppresor as reported by Alhasan in MCF-7 over expression studies [59]. CD97 and GPCR are two other direct targets of miRNA-126. The mechanism of tumor suppression is by down-regulating CD97 by binding directly to its 3′-UTR. CD97 is involved in invasion, migration and stimulates angiogenesis through binding integrin counter receptors on endothelial cells [60].

#### Plant Compounds Targeting VEGF Regualting miRNA

Several phytochemicals belonging to flavonoids, polyphenols, terpenoids, alkaloids are identified for their anticancer property which distinctly target VEGF and related factors in the signaling pathway [111] (Table 2). Cardamonin belonging to the flavonoid family has a number of pharmacological actions such as anti-inflammatory, anti-cancer and anti-oxidant properties [112]. Cardamonin exerts its anti-cancer potential by inhibiting proliferation, inducing apoptosis and can even reverse therapy resistance [113]. Its anti-angiogenic properties are well documented [114]. Cardamonin suppresses VEGF induced angiogenesis in a dose dependent manner by decreasing the phosphorylation of ERK and AKT. Reports indicate miRNA-21 is down-regulated within 24 h following 50 µM cardamonin treatment. Other AngiomiRs which are down-regulated include miRNA-23a, miRNA-132, miRNA-16 [114]. miRNA-21 is frequently up-regulated in some cancers and play a significant role in tumor angiogenesis. miRNA-21 is associated with poor prognosis in TNBC [115]. Flavonoids have a wide range of targets in both tumor cells and EC. Hence combination strategies with different phytochemicals are beneficial in controlling cancer growth and tumor angiogenesis. In a report published by Mirzaaghaei et al., investigating a plausible synergism between epigallocatechin-3-gallate (ECGC) and silibinin on EC and tumor cell, an up-regualtion of anti-angio miRNA-19b and down-regulation of angiogenic miRNAs in miR-17−92 cluster were observed [116]. Tumor suppressive property and other health benefits of resveratrol are very well documented. Anti-angiogenic property of resveratrol is exerted mainly by targeting the pro-angiogenic factors such as IL-8, CXCL8 and VEGF [117]. Resveratrol directly blocked VEGF signaling, decreased ROS production, suppressed eNOS and ERK1/2-AKT signaling. Different studies documented the regulation of various angiomiRs by resveratol which included miRNA-34a, miRNA-424, miRNA-503, miRNA-155. Among the alkaloids, brucine, evodiamine and matrine inhibit angiogenesis by targeting pathways suh as VEGF/AKT/NF-κB signaling [118,119,120]. However, the miRNA regulation of tumor angiogenesis by these alkaloids are rarely documented.

### 3.2. AngiomiRs in EC Metabolism

Angiogenesis is under the control of multiple factors. miRNA regulates cancer cell and EC metabolism by directly or indirectly targeting the genes regulating the expression of enzymes in the metabolic pathway. miRNA-153, miRNA-467, miRNA-126, miRNA-21 and miRNA-34a are angiogenic miRNAs responsive to high glucose level (hyperglycemia). These miRNAs are relevant in hyperglycemia induced cancer angiogenesis. EC are generally enriched with miRNA-126; it was noticeable in a study by Zampetaki et al. that the plasma sample profiling for miRNA in diabetes mellitus (DM) patients showed reduced level of miRNA-126. miRNA-155 is a key regulator in glucose metabolism in BC. miRNA-155 directly represses PIK3R1 and FOXO3a and demonstrates an activated glucose metabolism by up-regulating glucose transporters and metabolic enzymes hexokinase 2 (HK2), pyruvate kinase M2 (PKM2) and lactate dehydrogenase A (LDHA) [68].

#### Plant Compounds Targeting Metabolism Regulating miRNA

Phytochemicals modulate aberrant tumor metabolic pathways and metabolic reprogramming is an emerging strategy for controlling tumor growth. Tumor cells as well as TEC heavily depend on glycolysis (Warburg effect) for their energy requirements. Curcumin, an extensively studied polyphenolic compound, decreases glycolysis via dowregualting key glycolytic enzyme PKM2 via inhibiting mTOR/HIF-1α [121]. It up-regulates the PTEN expression via miRNA-21, thereby negatively regulating the PI3K/AKT pathway that regulates survival and glycolysis [122]. In addition, curcumin inhibits the expression of VEGFR 1/2/3. Resveratrol, a polyphenolic compound found abundantly in grapes, down-regulates oncogenic miRNA-155 that mediates the expression of GLUT genes and up-regulates miRNA-663 [123]. Betulinic acid (BA), a pentacyclic triterpene, has anti cancer properties and regulates glucose metabolism. BA suppresses metastasis in BC through β-catenin-mediated glycolysis. It inhibits proliferation and induces apoptosis via suppressing oncogenic miRNA-27a [124]. EGCG, another widely studied compound isolated from green tea, is a catechin with health benefits. Its anti-cancer properties were investigated in multiple types of cancers [125]. Very few studies have investigated the anti-glycolytic effect of EGCG in breast cancer cells [126]. Similar to curcumin and BA, EGCG also down-regulates two important angiomiRs, miRNA-21 and miRNA-27, that regulate glycolytic pathways. Genistein, a soy soflavone has negative regulatory effect on miRNA-155 [127]. Down-regulation of miRNA-155 decreases the glucose uptake and glycolysis via PI3K/AKT pathway by directly repressing PIK3R1 and FOXO3a. High expression of miRNA-155 in TNBC tumor specimens is indicated to have a positive correlation with glucose uptake [67,68]. Hence, targeting miRNA-155 by geinstein is an attractive anticancer strategy for BC treatment. Recent retrospective studies on Metformin, an anti-diabetic drug obtained from Galega officinalis, have demonstrated significant anti-cancer and anti-angiogenic properties. Its anti-angiogenic property is attributed to down-regualtion of miRNA-21 in EC by directly targeting phosphatase and tensin homolog (PTEN) and small-Mothers Against Decapentaplegic Drosophila Homolog Of 7 (SMAD7) [91].

### 3.3. AngiomiRs Responsive to Oxygen Level

As the tumor grows, the blood supply to the tumor becomes insufficient to meet the requirements for oxygen and nutrients. As a result, some regions of the tumor receive less blood supply, and usually the core becomes chronically hypoxic. In tumors, hypoxia brings changes in the tumor cell to acclimatize to stress by initiating angiogenesis, invasion and metastasis. This hypoxic response is largly mediated by HIF, a transcription factor that responds to low oxygen level. HIF-1,HIF-2, HIF-3 are the 3 isoforms present in humans, among which HIF-1 is highly expressed in tumors. HIF targeted genes include VEGF, glycolytic enzymes, glucose transpoters and insulin like growth factor (IGF) [128]. Earlier studies showed an altered miRNA profile in hypoxic condition. miRNA-153, miRNA-100, miRNA-182, miRNA-497, miRNA-155, miRNA-21, miRNA-20a are AngiomiRs that regulate angiogenesis via targeting HIF-1 in BC. HIF-1α is a down stream target of miRNA-21 acting via the AKT/ERK 1/2 pathway. Apart from this, miRNA-21a participates in the regulation of EC metabolism also. The expression of miRNA-153 is induced by hypoxia-induced ER stress in BC angiogenesis However, it has an anti-angiogenic action by down-regulating VEGFA secretion [70]. Hence miRNA-153 fine tunes HIF-1α/VEGFA axis in BC angiogenesis. Moreover, miRNA-153 has targets against several other oncogenes associated with survival (HECTD3) [129], EMT (MTDH) [71] and stemness (nuclear factor, erythroid 2 like 2 (NRF2)) [70]. The expression of miRNA-497 is deregulated in breast cancer cells in comparison to normal breast cell line. The expression of miRNA-497 was lower in hypoxic condition and higher in normoxic condition. VEGF was down-regulated in cells over-expressed with miRNA-497, hence VEGF and miRNA-497 showed a reciprocal effect [85]. Apart from being angiomodulator, miRNA-497 also contributes to EMT in breast cancer [86]. In view of different studies miRNAs can be either pro/anti angiogenic under hypoxic conditions.

#### Plant Compounds Targeting HIF-1α Regualting miRNA

Hypoxia activates transcription factor HIF which in turn activates the expression of many pro-angiogenic factors. Anti-angiogenenic strategies targeting HIF signaling pathway act as an attractive approach for anticancer therapy. Wogonin (flavone), triptolide and EGCG are potent inhibitors of HIF-1α in both tumor cell and EC [130,131,132]. In contrast, angiogenesis studies conducted in EC showed stabilization of HIF-1α and over expression of its target gene VEGF under normoxia following quercetin (flavonol) treatment [133]. Hence its health implication on diet rich in quercetin cannot be overlooked in cancer pateints. Though many phytochemicals were investigated for its anti-angiogenic property, their role in miRNA regulation of HIF-1 signaling is easy to overlook.

### 3.4. ROS Sensing miRNAs and Tumor Angiogenesis

Reactive oxygen species (ROS) are endogeneously produced by mitochondria during aerobic metabolism, but they have a controversial role in tumor development. In tumors, ROS have multiple biological effects and a growing body of literature highlights the role of miRNA in sensing ROS and their implication in cancer [134]. ROS can positively or negatively regulate the miRNA expression and exert their biological effect through the regulation of down stream signaling involving PI3K/AKT pathway, MAPK pathway and NF-κB pathway [135]. ROS promotes angiogenesis by stabilizing HIF-1α, and activates EMT, invasion and metastasis. Studies confirm the presence of high levels of ROS in breast cancer tissue compared to normal breast tissue. Moreover, it is interesting to note that different subtypes of BC have different levels of ROS production among which TNBC shows the highest level of ROS [136]. A recent review by Babu and Tay on ROS signaling in cancer progression mentions an excisting crosstalk between ROS and miRNA regulation [137]. ROS can affect the miRNA expression by different mechanisms, such as epigenetic modulation of miRNA or by modifying the expression of transcription factors involved in the miRNA biogenesis. Reciprocally, miRNA regulates the endogeneous production of ROS by directly targeting the genes involved in ROS production or synthesis of anti-oxidants. In BC, ROS levels are linked to the expression of miRNA-28, miRNA-210 [138], and in turn intracellular ROS can modify the expression of several angiomiRs important in BC cancer progression (e.g., miRNA-21, miRNA-145 and miRNA-34a). Earlier we discussed the role of miR526b/miR655 in tumor angiogenesis, invasion and metastasis in BC. Furthermore, its role in oxidative stress in BC was recently investigated by Shin et al. [139]. They reported that thioredoxin Reductase 1 (TXNRD1) an oxidoreductase is overexpressed in both MCF-7 and HUVEC cells when grown in cell free conditioned media containing miRNA-526b/miRNA-655. Mechanisticaly miRNA-526b/miRNA-655 down-regulates two inhibitors of TXNRD1, (i.e., TCF21 and PBRM1). Highly metastatic BC cell lines (MCF7-COX2, Hs578T and MDA-MB-231) show maximum upregulation of TXNRD1 and parallely high levels of miR526b/miR655 expression [139]. In a study performed in HUVECs under high glucose condition, a higher expression of the glucose sensing miRNA-21 was observed, regulating the ROS production via KRIT1 pathway, an endogeneous regulator of endothelial ROS homeostasis [140]. High levels of miRNA-21 expression clinically correlate with the advanced stage of breast cancer, metastasis and poor prognosis [141].

#### Plant Compounds Targeting ROS Sensing miRNAs

Bioactive compounds such as ascorbic acid, gallic acid, quercetin, caffeine have antioxidant properties. Various epidemiological studies show health benefits of dietry intake of food rich with high anti-oxidants. However, phytochemicals can have pro- or anti-oxidant activity, and the same compound can exhibit both properties at different concentrations. For example, quercetin is reported to have anti-oxidant activity at lower concentration (0.1–20 μM) while showing pro-oxidant activity at a higher dose (> 50 μM) in relation to glutathion concentration and super oxide dismutase activity as tested in A549 cells [142].

## 4. Clinical significance of miRNA in BC

Breast cancer is the most common gynecological cancer leading to nearly 15% of cancer related death in women [160]. Molecular analysis of breast cancer based on the gene expression profile (e.g., estrogen receptor (ER), progesterone receptor (PR), human epidermal growth factor receptor 2 (HER2)) has enabled the classification of BC into different subtypes, such as luminal A, luminal B, HER2 and basal (or triple negative BC) types of breast cancers. This classification has led to the identification of biomarkers which are helpful for diagnosis, prognosis and prediction of the therapeutics. Similarly, miRNA profiling of BCs took BC research to a more advanced level where it is helpful to classify BCs in more specific entities and thus enhance the capability to predict the recurrence, metastasis and response to therapy, and identify possible chemotherapeutic resistance. For instance, increased expression of miRNA-21 identifies advanced BC, lymph node metastasis and poor prognosis [141,161]. miRNA-93 is associated with lymph node metastasis and is relevant in basal subtype of BC [61,162]. Similarly, miRNA-155 showed increased expression in late stage and aggressive TNBCs with low levels of VHL indicated poor prognosis [67]. miRNA-153 is down-regulated in breast cancers and hence has a tumor suppressor role [71]. Expression of miRNA-153 negatively correlated with metadherin, an oncogene which enhances invasion via PI3K/Akt, and Wnt/beta-catenin signaling pathways. Clinical data from BC patients undergoing a TAC regime (docetaxol, doxorubicin plus cyclophosphamide) demonstrated a strong correlation with miRNA-205 expression [57]. In ER and PR positive BC, the expression of miRNA-182 up-regulation was correlated with FBXW7 down-regulation. Here, miRNA-182 have shown to promote HIF-1α expression [83]. A possible link between miRNA-497 and breast cancer progression was identified by Wu et al. by demonstrating an inverse correlation with Slug, a protein associated with EMT [86]. An important angiogenic pathway VEGF/PI3K/AKT signaling was found activated in BC specimen with low miRNA-126 levels [89]. miRNA-105 is indicated in pre-metastatic stage, hence useful as a marker for early stage detection [64]. Each BC subtype shows a different preference of site of metastasis, and so does the miRNA expression related to metastasis [163]. The advances in miRNA research should enable a more precise targeting of genes using less invasive procedure for evaluation by examining the levels of circulating miRNA. In vitro studies showing the restoration of gene function by over expression or inhibition of dysregulated miRNA during the tumorigenesis identifies miRNA as suitable target for anticancer therapy. Identification of more unknown miRNAs and their clinical correlation in breast cancer progression is necessary for effective breast cancer management.

## 5. Discussion and Concluding Remarks

Research on natural compounds as potential source of anti-cancer drugs is generating considerable interest to invest more in plant-based research. Mechanistic studies evaluating therapeutic potential of plant based compounds reveal multi targeted action on the target cell [164]. The principle behind any therapeutic approach against tumor growth is essentially by targeting the hallmarks of cancer, miRNA profiling of different cancers reveals a striking association of miRNA with all stages (cancer hallmarks) of the cancer progression. It is interesting to note that miRNA related studies are gaining importance as potential biomarkers, aiding in prognosis and diagnosis of cancers. Its scope now has extended to its use in targeted therapy. In light of the recent researches natural compounds directly or indirectly regulate miRNAs and control cancer growth [165]. Evidences support the epigenetic silencing of various miRNA relevant in cancer. Plant derived natural compounds have multiple biological effects among which epigenetic modification is gaining considerable attention as an effective preventive and treatment strategy for cancer [166]. miRNA can regulate the epigenetic mechanism by modifying expression of enzymes methyltransferases and histone deacetylases and, vice versa, epigenetic mechanism can regulate miRNA expression. Dietary polyphenols have shown to modify altered epigenetic mechanism by turning on the expression of miRNA regulating tumor suppressor genes which were silenced epigeneticaly in cancers [167].

The hypothesis of targeting angiogenesis for suppressing tumor growth was first reported in one of the pioneer studies by Folkman et al. [168]. According to Folkman, drugs used for anti-angiogenic therapy include inhibitors targeting EC directly by down-regulating VEGF, bFGF, its receptors, HIF-1α and up-regulating thrombospodin 1, maspin, HIF-1α inhibitor and TIMP2. Among the prominent cell signaling in EC, hypoxia induced HIF-1α/VEGF stands central to switching of a quiescent EC to an active EC phenotype. In the context of tumor angiogenesis, AngiomiRs regulates angiogenic process both negatively and positively by regulating the function of the above mentioned pro-angiogenic factors, while in tumors this balance is lost, which results in the formation of defective tumor vasculature. Several studies emphasize the central role of angiogenesis in BC development and confirm the prominent role of angiogenesis in human invasive BC [12]. Breast cancers which are classified based on the hormone receptor status and molecular profile [169] have differentially regulated miRNA profiles [170]. The miRNA profile of different subtypes of BC show clear correlation with the clinicopathological factors such as grade, stage, Tp53 status and vascular invasion [170].

miRNA targets multiple genes, or a single gene can be regulated by multiple miRNAs. Multiple pro-angiogenic factors, mainly VEGF, are up-regulated in invasive breast cancer cases, and numerous miRNAs have direct targets on several pro-angiogenic factors, making them attractive targets for anti-angiogenic therapy. Furthermore, miRNA can enhance drug interactions as stated by Baldassari et al. where they report miRNA-126 (EC specific) as modulator of CDK4/6 and PIK3CA inhibitors [171]. From the evidence, it is obvious that drugs modify the expression of miRNA and miRNA, and in turn can modulate drug efficacy and chemoresistance [171,172]. Many plant compounds were identified for their anti- or pro-angiogenic potential, their angiogenic action depending on the type of compound and concentration. Some of the compounds reviewed here show distinct regulation of miRNA expression by phytochemicals. Clinically relevant, miRNA-21 (whose down-stream target is HIF-1α), which has been implicated in advanced BC, lymph node metastasis and poor prognosis, is down-regulated by phytochemicals such as cardamonin, curcumin, metformin and EGCG. Interestingly, resveratrol up-regulated the expression of this oncogenic miRNA-21. However, other oncogenic-miRNA were suppressed by resveratrol.

Metabolic shift in cancers was overlooked until Otto Warburg discovered the glycolytic phenotype of cancer cells. This metabolic shift contributes to excess glycolytic flux, lactate, low pH, activation of oncogenes and suppression of tumor suppressor genes. Glycolytic switch in cancer cells together with tumor secreted growth factors and TME drives angiogenesis in tumors by reprogramming EC metabolism and angiogenic signaling in EC [173,174]. miRNA which are angiogenic also participate in the regulation of EC metabolism. For example, miRNA-93, miRNA-155, miRNA-153, miRNA-467, miRNA-23a, miRNA-126, etc. modulate EC metabolism. Compounds such as luteolin, genistein and EGCG exert an anti-glycolytic effect by modulating these miRNAs. Targeting the metabolism is a new approach for anti-tumor strategy as down-stream signaling of HIF-1α/VEGF signaling converges at both metabolism and proliferation signaling in EC [175]. The anti-diabetic drug metformin showed reduced cancer risk and improved patient survival in all types of cancer patients with type II diabetes. In vitro and in vivo data show significant inhibition in cell proliferation in TNBC [150]. In contrast to the above observation, Bakhashab et al. reported pro-angiogenic effects of metformin in HUVEC under hyperglycemia/hypoxia conditions where they observed increased migration and decreased apoptosis via up-regulation of VEGFR 1/2 signaling [176].

In view of the literature reviewed, there are ample studies indicating the role of phytochemicals in 1) modulating miRNAs in cancer [39,177] and 2) inhibition of tumor angiogenesis in different cancers [178]. However, there are very few studies that directly link all three key aspects (phytochemicals, miRNA and anti-angiogenesis) that we have considered in this review. Additionally, internet searches performed in ClincalTrials.gov (database for registered clinical trials), using the key words phytochemicals/miRNA/anti-angiogenesis, yielded little information indicating that the possibility of targeting anti-angiogenic miRNAs using phytochemicals in the treatment of cancers has not been explored at the clinical level. Therefore, more clinical studies/trials are warranted in this area to the extend the knowledge and data available from cell based and in vivo experiments to a clinical scenario. Hence, there is a significant scope for further research in phytochemical targeting of miRNA to develop it as a potential anti-cancer strategy. However, selected phytochemicals can have dual effect on angiogenesis, where results should be interpreted and translated meaningfully for each type of cancer.

## Figures and Tables

**Table 1:** Detailed overview of miRNAs involved in tumor metabolic and angiogenic signaling.

	miRNA	Cellular Function	Target	Signaling Pathway	Cell Line	References
**1**	miRNA-105	Promote metastasis	ZO-1 (tight junction protein) MXI1	miRNA based metastasis MYC pathway	MDA-MB-231	[61,62,63,64]
**2**	miRNA-93	EC glycolysis and EC proliferation	KLF2 and PFKFB3 (in glycolysis). FOXO1 and MYC (in proliferation). WNK lysine deficient protein kinase 1 (WNK1) ↓LATS2	Glycolytic pathway and VEGF pathway Enhances angiogenesis and metastasis to the lungs	MT-1 MDA-MB-231 Breast carcinoma specimens.	[25,65]
**3**	miRNA-10b and miRNA-196b	Produced in response to tumor secreted VEGF and regulate EPC function and angiogenesis	↓HOXD10	HOX pathway	IDC grade III tumors	[66]
**4**	miRNA-155	Up-regulates glucose transporters and glycolytic enzymes. Associated with poor prognosis and metastasis.	↓VHL	Oncogenic ↑HIF ↑PIK3R1-PDK/AKT-FOXO3a-cMYC axis	TNBC- late-stage (stage III/IV), lymph node metastasis	[67,68]
**5**	miRNA-4530	Decreases proliferation, induces apoptosis, promotes angiogenesis	↓VASH1 (endogenous angiogenesis inhibitor	Arrest at S/G2	MCF-7 MDA-MB-231 HUVEC	[69]
**6**	miRNA-153	Response to high glucose Tumor suppressor EMT suppressor Down-regulated in BC	IRE1α-XBP1 ↓MTDH	↓HIF-1α/VEGFA signaling TGF-β-mediated-EMT	MDA-MB-231 HCC1937 Luminal A and B, Basal, HER 2 positive	[70,71]
**7**	miRNA-205	Enhances chemosensitivity of breast cancer cells to TAC chemotherapy (docetaxol, doxorubicin plus cyclophosphamide)	↓VEGFA and FGF2	Tumor suppressor, ↓PI3/AKT signaling pathway	MCF-7/A02 and CALDOX	[57]
**8**	miRNA-206	Suppresses glycolysis	↓VEGF PFKFB3	VEGF/MAPK3/SOX9 ↓proliferation and metastasis	MDA-MB-231, MDA-MB-435, and HCC1395	[72,73]
**9**	miRNA-221/222	Anti-angiogenic Anti-proliferative Increases senescence Response to high glucose	↓eNOS ↓ZEB2 ↓P27	eNOS signaling	Mouse microvascular endothelial cells (MMECs)	[74,75]
**10**	miRNA-100	Mesenchymal stem cell derived exosomes	↓VEGF	↓mTOR/↓HIF-1/VEGF pathway	MDA-MB-231, MCF-7, T47D, HUVEC BM-MSC	[76]
**11**	miRNA-29b	Invasion, proliferation and migration	AKT3 SPIN1	AKT3/VEGF/C-myc ↓WNT and AKT	HUVEC MDA-MB-231	[77]
**12**	miRNA-23a	Up/down-regulated in specific type of cancers. Aerobic glycolysis Anti-angiogenic	↓LDHA and LDHB	Glycolytic pathway miR-23a/↓RUNX2/↓VEGF-A	HUVEC	[78,79]
**13**	miRNA-23b	Inversely correlated with metastasis	↓PAK2, MLC II Phosphorylation, under the regulation of AP-1, directly target cytoskeleton genes. JAM-C and ZO-2	Both oncogenic and tumor suppressive Cytoskeletal re-organization, migration and metastasis ↑Vascular permeability and EC tube formation	MCF7 MDA-MB-231 HUVEC Xenograft	[80,81,82]
**14**	miRNA-182	Oncogenic	↓FBXW7	Hypoxia/miRNA 182/↑HIF-1α/VEGFA axis	hy926 MCF-7 ER and PR positive BC	[83]
**15**	miRNA-497	Down-regulated EMT	↓VEGFR2, ↓VEGF and ↓HIF-1α ↓ Slug	VEGFR2/Raf/ERK/MEK pathway VEGFR2/PI3K/AKT pathway Tumor-suppressor Hypoxia/miR-497/HIF-1α pathway	MCF-7 4T1 Xenograft Invasive ductal breast cancer	[84,85,86]
**16**	miRNA-140-5p	Tumor suppressor	↓VEGFA, ↓MMP9	Proliferation-↓Ki 67 Angiogenesis- ↓VEGFA Metastasis- ↓MMP-9	MCF-7 MDA-MB-231	[11]
**17**	miRNA-467	Response to hyperglycemia Tissue specific	↓TSP-1	Pro-angiogenic	EMT6 (mu) C116	[87,88]
**18**	miRNA-126	Endothelial cell specific	↓VEGFA ↓PIK3R2 ↓SPRED1	VEGF/PI3K/AKT	MCF-7	[89]
**19**	miRNA-27a	Pro-angiogenic	↑ZBTB10	Autocrine VEGF/RUNX1/miR27a/ZBTB10 signaling loop	MDA-MB-231 BCSLCs (SK-3rd)	[90]
**20**	miRNA-21	Responsive to glucose level Pro-angiogenic Modulates ROS level Promotes metastasis	↓PTEN and SMAD7 HIF-1α PDCD4, maspin LZTFL1	TGF-β, AKT-, SMAD- and ERK-dependent signaling Oncogenic Decreased apoptosis and increased proliferation Metastasis and invasion	MCF-7 MDA-MB-231	[91,92,93,94,95,96,97,98]
**21**	miRNA-503	Anti-angiogenic	↓CCND1	Tumor suppressor ↓FGF2 and VEGFA (hepato cellular carcinoma) Down-regulated in BC tissue	MCF-7, T47D, MDA-MB231, BT549, SKBR3, ZR-75-30	[99,100]
**22**	miRNA-34a	Response to high glucose	↓SIRT1	Suppress proliferation and invasion by targeting Notch Inhibits BC stemness Tumor-suppressive role	BT-474, MDA-MB-231, MDA-MB-435, MDA-MB-468, SK-BR-3, EC	[101,102,103]
**23**	miRNA-26a	↓ VEGF dependent migration and proliferation Anti-angiogenic	↓NgBR MCL-1	VEGF/NgBR/↓eNOS pathway Tumor suppresor	HUVECs MDA-MB-231, MCF-7, MDA-MB-435, MDA-MB-468	[104,105]
**24**	miRNA- 98	Anti-angiogenic	↓MMP11, ↓ALK4	Inhibits cell spreading, cell invasion and tubule formation. Suppress proliferation and survival of BC cells	4T1, MT1, MDA-MB-231, MDA-MB-468, Xenograft	[106]
**25**	miRNA-126	Response to high glucose ↓Type 2 diabetes	↓VEGFA and PI3K regulatory subunit 2 (PIK3R2) CD97, GPCR	VEGF/PI3K/AKT Tumor suppressor EC migration and tumor angiogenesis	MDA-MB-231 MCF-7 plasma from DM patients	[59,60,107]
**26**	miRNA-145	Anti-angiogenic	N-RAS and VEGF-A IGF-I/IRS	PI3/AKT/mTOR/p70S6K1 Tumor suppressor Suppressed the invasion and tube formation in EC IRS1/N-RAS/VEGF pathway	MCF-7 MDA-MB-231	[108]
**27**	miRNA-20a	Predominantly in TNBC pro-angiogenic	↑VEGFA and HIF-1α	VEGFA dependent angiogenesis	MCF-7 MDA-MB-231	[109]
**28**	miRNA-526b miRNA-655	Tumor associated angiogenesis and lymphangiogenesis	EP4, ↓PTEN and PI3K/Akt ↑VEGFA	PI3K/AKT pathway	MCF-7 ER and PR positive HER 2 negative BC	[46]
**29**	let-7a	Regulates key anabolic enzymes ROS production	Stearol Co-A Desaturase (SCD) G6PD, FASN, BACH1	Glycolytic pathway OXPHOS pathway Sensitizes BC to doxorubicin	MDA-MB-231	[110]

**Table 2:** Phytochemicals and their target miRNAs regulating tumor angiogenesis.

	Compound	Target miRNA	Effects	Cells	References
**1**	Cardamonin (50 μM)	↓miRNA-21	↓VEGF mediated angiogenesis, inhibits EC proliferation and migration	HUVECs	[114]
**2**	Resveratrol (50 μM)	miRNA-34a miRNA-424 miRNA-503 ↓miR-155 ↑miRNA-21 ↑miR129 and miR489 ↑miR-141 and miR-200c	↓VEGF-↓glycolytic genes, ↓ERK 1/2, ↓NO ↓IL-8/CXCL8 ↓DNMT1, DNMT3b ↓Stemness	HUVEC, Estrogen dependent mammary carcinoma rat model MDA-MB-231	[117,123,143,144]
**3**	Silibinin (in combination with EGCG) (91.22 μM and 68.07 μM)	↓miRNA-21 ↓miR-17−92	↑CASP-9 and APAF-1 ↓VEGF−VEGFR2 axis	T47D, HUVEC	[145]
**4**	Curcumin (30–60 lmol l^−1^)	miRNA-29 ↓miRNA-21 ↑miR-15a and miR-16	PDCD4, PTEN/PI3K/AKT and NF-κB ↓Bcl-2 Pro- or anti-angiogenic at different concentrations	MCF-7	[146,147,148]
**5**	Metformin (anti-diabetic drug) (20 mM)	↓miRNA-21 ↓miRNA-221 miRNA-34a ↑miRNA-26a ↓Let-7a	Anti-angiogenic via ↓TGF-β, PTEN, EHZ2 and SMAD7 eNOS signalling	HUVECs MDA-MB-231, MDA-MB-468, BT 549, MCF-7	[75,91,101,149,150,151]
**6**	Genistein (10–25 μM)	↓miRNA-155 ↑miRNA-23b ↓miRNA 221/222	↑FOXO3, PTEN Regulates viability and apoptosis via transcriptional regulation of miR-155 ↓Metastasis, enhances focal adhesion connections	MDA-MB-435, Hs578t	[127,152]
**7**	EGCG (20 µM)	↓pro angiogenic ↑anti angiogenic miRNA ↓miRNA 27a ↓miRNA-21	↓HIF1α, GLUT1 ↓HK, PFK, LDH (glycolytic enzymes) ZBTB10	4T1, MCF-7	[126] [116,153]
**8**	Luteolin (50 µmol/L)	↓miRNA-155 ↓miRNA-21 ↑miR-34a ↑miRNA-181a, ↑miRNA-139-5p ↑miRNA-224 and ↑miRNA-246 ↑miRNA-203	↓Notch signaling, ↓PI3K/Akt, ↓VEGF, ↓Notch-1, ↓Hes-1, ↓Hey, ↓VEGF, ↓Cyclin D1 and MMP2/9 ↓Tumor growth, ↓Invasion ↓Angiogenesis Anti-oxidant	MDA-MB-231, HUVECs	[154,155,156]
**9**	Sinomenine (4 µM)	↑miRNA-29	miR-29/PDCD-4 axis ↓Tumor growth, ↓Metastasis, invasion, vascular normalization, improved tumor immunity	HUVEC 4T1(murine breast cancer model) MDA-MB-231, MCF-7	[157,158]
**10**	Triptolide (15 ng/mL)	↑miRNA-146a	↓Rho GTPase - ↓Metastasis and invasion ↓ERK1/2-/HIF-1α/6666VEGFA axis	MDA-MB-231	[132,159]
**11**	Betulinic acid (2.5–10 μmMol/L)	↓miRNA-27a	↑ZBTB10 and Myt-1	MDA-MB-231, BT-549	[124]

## Abbreviations

ALK4	Activin receptor-like kinase 4
ASCT2	System ASC amino acid transporters 2
BACH1	BTB Domain And CNC Homolog 1
bFGF	Basic fibroblast growth factor
BM-MSC	Human bone marrow-derived mesenchymal stem cell
CPT1A	Carnitine palmitoyl transferase 1A
CXCL8	Chemokine (C-X-C motif) ligand 8
DNMT	DNA methyltransferase
EMT	Epithelial–mesenchymal transition
EPC	Bone marrow–derived endothelial progenitor cells
FA	Fatty acid
FASN	Fatty acid synthase
FATP	Fatty acid transporter protein
FGF2	Fibroblast grow factor-2
FZD	Frizzled receptor
G6PD	Glucose-6-phosphate dehydrogenase
GPCR	G-protein-coupled receptor
HIF	Hypoxia inducible factor
IDC	Infiltrating ductal carcinoma
IGF-I	Insulin-like growth factor I
IRS1	Insulin receptor substrate 1
LAT1	L-type amino acid transporters 1
LATS2	Large tumor suppressor, homology 2
LDHA/B	Lactate dehydrogenase A and B
Let-7a	Lethal-7a
LZTFL1	Leucine zipper transcription factor-like 1
MLC II	Myosin light chain II
MMP11	Matrix metalloproteinase 11
MMP9	Matrix metalloproteinase 9
MTDH	Metadherin
NgBR	Nogo-B receptor
NO	Nitric oxide
PDCD4	Protein programmed cell death 4
PFKFB3	Phosphofructokinase-2/fructose-2,6-bisphosphatase
TIMP2	Tissue inhibitor of metalloproteinases 2
TME	Tumor microenvironment
TNFα	Tumor necrosis factor α
VASH1	Vasohibin-1
WNK1	WNK lysine deficient protein kinase 1
ZO-1	Zonula occludens 1
α KG	α keto glutarate
